# Loss of *CDKN2A/B* is a Hallmark of RTK II Glioblastomas

**DOI:** 10.7150/jca.122609

**Published:** 2026-01-01

**Authors:** Celina K. Langwieder, Dorothee Hölzl, Georg Hutarew, Hans U. Schlicker, Beate Alinger-Scharinger, Christoph Schwartz, Karl Sotlar, Theo F. J. Kraus

**Affiliations:** 1 Institute of Pathology, University Hospital Salzburg, Paracelsus Medical University, Müllner Hauptstr. 48, A-5020 Salzburg, Austria.; 2 Department of Neurosurgery, University Hospital Salzburg, Paracelsus Medical University, Ignaz-Harrer-Str. 79, A-5020 Salzburg, Austria.

**Keywords:** glioblastoma, *CDKN2A/B*, DNA methylation analysis, RTK I, RTK II, MES

## Abstract

Glioblastomas represent the most prevalent primary brain tumors in adults. Due to their highly malignant biological behavior, they are classified as grade 4 according to the World Health Organization (WHO) classification of brain tumors. Despite the progress in understanding the molecular pathogenesis of these tumors, no curative therapy has been developed for patients with glioblastoma. In this study, an integrated comparative analysis of *cyclin-dependent kinase inhibitor (CDKN) 2A/B* chromosomal deletion was performed on 45 glioblastomas, representing the most frequent molecular subtypes of glioblastomas, receptor tyrosine kinase (RTK) I (n=13), RTK II (n=15), and the mesenchymal subtype (MES) (n=17). The analysis of copy number variation (CNV) profiles was conducted on *CDKN2A/B* losses. Subsequent statistical analysis was then applied to correlate the collected data with molecular glioblastoma epigenotypes. Loss of *CDKN2A/B* was found 44% (20/45) of all glioblastomas, thereby, in 46% (6/13) of RTK I, 67% (10/15) RTK II, and 24% (4/17) of MES. Statistical analysis showed that loss of *CDKN2A/B* is significant (p < 0.01) in RTK II compared with MES. Even though *CDKN2A/B* does not per se function as a molecular target, there is great potential for enhancing treatment outcomes through the restoration of the tumor-suppressing capabilities of *CDKN2A/B*. This strategy can be employed in therapeutic interventions and is a promising avenue for research. This efficacy of this approach demonstrates high potential, as evidenced by its efficacy in other tumors, including melanoma.

## Introduction

According to the 2021 World Health Organization (WHO) classification for central nervous system (CNS) tumors, the integration of both morphology and molecular markers is identified as an essential aspect in advanced brain tumor diagnosis. 1 According to the prevailing molecular taxonomy, diffuse gliomas in adults are classified into three primary categories: astrocytomas, oligodendrogliomas, and glioblastomas: 1 The screening of mutations present within the isocitrate dehydrogenase (*IDH*) 1 and 2 genes is imperative for the accurate diagnosis of an astrocytoma. The presence of *IDH1* and *IDH2* mutations, in combination with losses of chromosome 1p and 19q, serves as a distinguishing characteristic in the diagnosis of oligodendroglioma. 1 Diffuse gliomas without *IDH* mutations are classified as glioblastomas. 1 Therefore, the degree of anaplasia, as reflected by the presence of mitoses, microvascular proliferation, and necrosis, is reflected by the addition of a central nervous system (CNS) World Health Organization (WHO) grade. IDH-mutated astrocytomas are classified as CNS WHO grade 2 to 4, while IDH-mutated 1p/19q co-deleted oligodendrogliomas are classified as CNS WHO grade 2 to 3. Notably, all IDH wildtype glioblastomas are classified as CNS WHO grade 4, indicating their particularly aggressive nature. 1

Glioblastomas IDH wildtype are the most malignant brain tumors in adults 1 and with 3-4 reported cases per 100,000 population also the most frequent primary brain tumor of adults in the western world. 1 The initial discovery of O6-methylguanine-DNA methyltransferase (*MGMT*) promoter methylation in 2008 signaled a new era in the field of glioblastoma therapy, offering novel therapeutic prospects: 2-4 Hypermethylation of the *MGMT* promoter has been demonstrated to be associated with significantly prolonged survival in patients who receive adjuvant radio-chemotherapy with temozolomide according to the EORTC/NCIC protocol. 5 Besides the addition of adjuvant tumor treating field therapy, till date, no significant progresses in glioblastoma therapy have been made. 1

Glioblastomas can be molecularly subclassified with the most frequent molecular tumor subtype receptor tyrosine kinase (RTK) I, RTK II, and mesenchymal (MES). 1 RTK I glioblastomas show increased amplification of *platelet derived growth factor receptor A* (*PDGFRA*), while RTK II glioblastomas are enriched with amplification of *epidermal growth factor receptor* (*EGFR*) gene, and MES glioblastomas show no typical recurring alterations. 6-8 Clinically, patients with RTK II glioblastomas show a significantly higher incidence of seizures compared to those suffering from tumors of RTK I and MES subtypes. 9

A promising novel approach for individualized patient care is the tumor suppressor gene *cyclin dependent kinase inhibitor* (*CDKN*) *2A/B* and its reconstitution as a tumor-suppressor. 10 *CDKN2A* gene is frequently inactivated in malignancies. 10 This gene encodes for the two tumor suppressor genes p16^INK4a^ and p14^ARF^, respectively, and is located next to *CDKN2B* that acts as multiple tumor suppressor 2 (MTS2, p15^INK4b^). 10 *CDKN2B* functions as a cell-cycle regulator that exerts inhibitory effects on the cyclin-dependent kinases CDK4 and CDK6. 11 Its significance is accentuated in the absence of p16^INK4a^, where it collaborates with oncogenes to induce tumors in specific cell types. 11 Analysis of p15^INK4b^ reveals that it functions as a significantly more potent tumor suppressor compared to p16^INK4a^, through its inhibition of both cell cycle and aerobic glycolysis. 11

Inactivation of the gene group *CDKN2A/B* is a common phenomenon in cancer such as in melanoma, and gene inactivation has been reported to occur in the range of 40% to 70%. 10 The loss of the tumor suppressor gene *CDKN2A* is a frequent occurrence that, for example, contributes to melanoma progression. 10 Consequently, therapeutic strategies targeting and reconstituting *CDKN2A* loss hold considerable promise for enhancing melanoma treatment outcomes. 10 A notable example of such a strategy involves the pharmacological inhibition of p16, which targets CDK4/6. 10

Alterations of *CDKN2A/B* have also been observed in the context of glioma. Homocygote deletions of *CDKN2A/B* have been identified in both low-grade and high-grade gliomas. 12 The majority of studies were conducted on IDH mutated gliomas, which show an association with a more unfavorable prognosis. 13-20 Several studies have also been conducted on *CDKN2A/B* loss in IDH wildtype glioblastoma. 21-25 These studies suggest that the deletion status of CDKN2A/B may be associated with overall survival and the efficacy of therapy in specific subpopulations. 21-24, 26 Nonetheless, these studies did not consider the various molecular subtypes of glioblastoma. 21, 23, 26

In this study, an integrated analysis of *CDKN2A/B* chromosomal deletion was performed in 45 glioblastomas with IDH wildtype, and the results were correlated with the molecular subtypes of RTK I, RTK II, and MES.

## Materials and Methods

### Tissue collection

A total of 45 anonymized tissue samples were analyzed. These samples contained glioblastomas IDH wildtype of CNS WHO grade 4 and the following molecular subgroups: RTK I (n = 13), RTK II (n = 15), and MES (n = 17). The samples were classified according to the 2021 CNS WHO classification. 1 All tumor samples were stored in the archives of the University Institute of Pathology of the University Hospital Salzburg. The samples used in this study have been formalin fixed and paraffin embedded (FFPE) tissues. Prior to study inclusion, samples were anonymized according to the local ethical guidelines.

### Molecular genetic characterization of gliomas

Molecular genetic analysis of glioma samples was performed as previously described. 27 In brief, representative tumor tissues with at least 90 % of viable tumor cells were microscopically identified. 27-30 DNA extraction was performed applying the Maxwell system (Promega) according to the manufacturer's instructions. 27 Mutational analysis of *telomerase reverse transcriptase* (*TERT*) was performed using Sanger sequencing, *IDH1* and *IDH2* genes was performed with the AmpliSeq for Illumina Cancer Hotspot Panel v2 (Illumina) or the AmpliSeq for Illumina Focus Panel (Illumina), respectively, on an Illumina MiniSeq next generation sequencing device following the manufacturer's protocols. 27

### Infinium methylation EPIC array analysis

Methylation analysis of glioma samples was performed using the Infinium Methylation EPIC Bead Chip (Illumina) according to manufacturer's protocol. 31-33 Raw data (idat-files) were analyzed using the molecularneuropathology.org bioinformatics pipeline of the German Cancer Research Center (Deutsches Krebsforschungszentrum, DKFZ) and the current brain tumor classifier 34, while CNV analysis is an integrated part of the pipeline. Loss of *CDKN2A/B* was assessed using the generated CNV plots and ImageJ. *CDKN2A/B* status was interpreted in accordance to Stichel *et al.*: If the respective probes showed a decreased intensity of less than -0.6 on the log2-scale from the CNV after baseline correction (relative probe intensity), chromosomal loss was assumed. 35

### Statistical analysis

Statistical analysis was performed using Prism 9 (GraphPad) software suite and Microsoft Excel applying Student's t-test. Statistical significance was assumed for p-values < 0.05.

## Results

### Detection of *CDKN2A/B* loss applying Infinium EPIC methylation bead chip analysis

**Figure 1:**
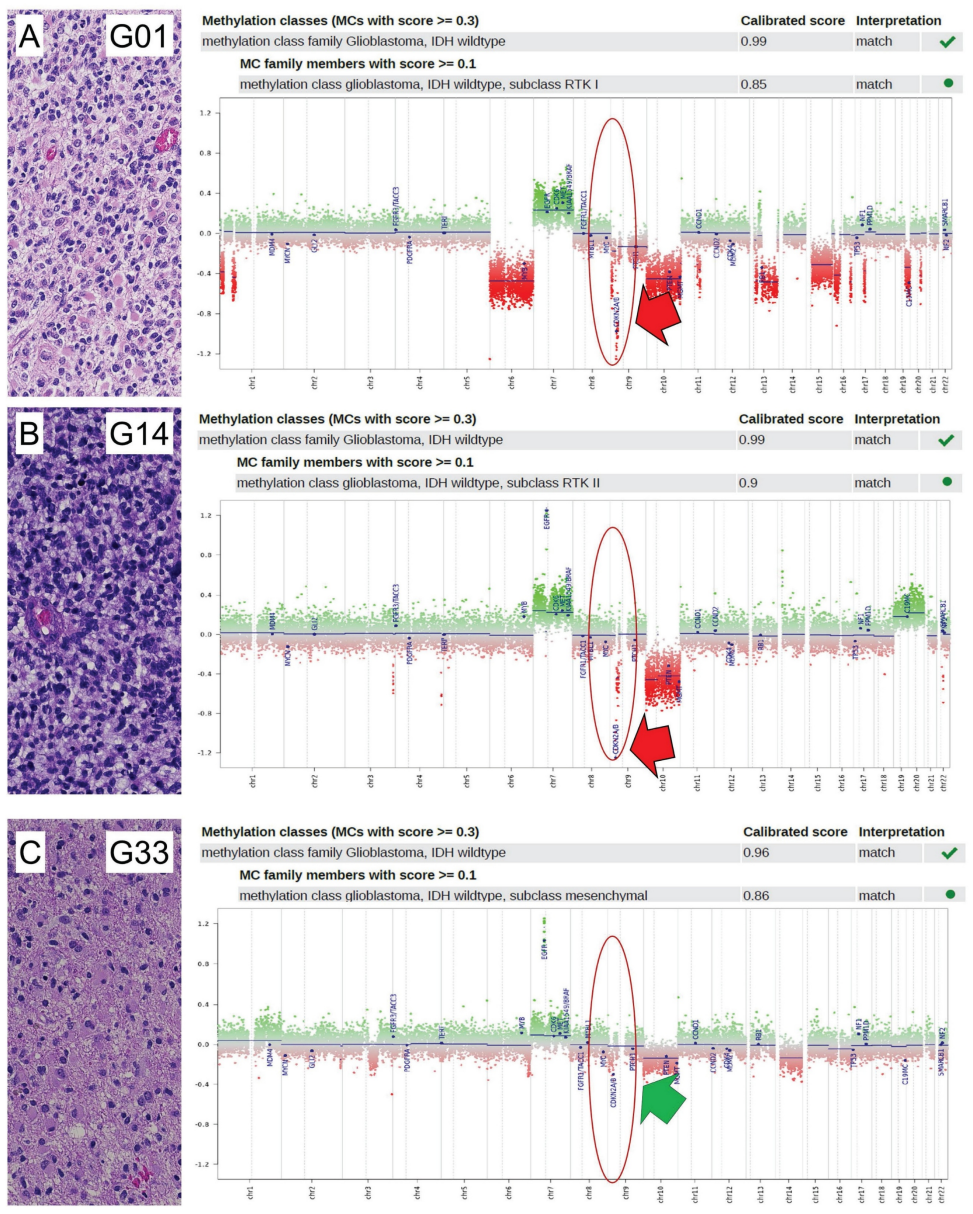
** Detection of CDKN2A/B loss in glioblastomas.** Analysis of 45 glioblastoma samples on CDKN2A/B status was determined by analysis of CNV plots of Infinium methylation EPIC bead chips. If the relative probes showed a decreased intensity of less than -0.6 on the log2-scale from the CNV after baseline correction (relative probe intensity), CDKN2A/B was regarded as lost, otherwise as retained. Shown are three exemplified pictures (case G01, G14, and G33) of glioblastoma of RTK I (A), RTK II (B), and MES subtype (C). In each case, histology in HE (20x magnification), allocation to the molecular subgroup and CNV plot are indicated. Case G01 (A) and case G14 (B) showed loss of CDKN2A/B (indicated by a red arrow), while case G33 (C) showed retained CDKN2A/B (indicated by a green arrow). Locations of CDKN2A/B are indicated by red circles.

In this study, we conducted a comprehensive profiling analysis of loss of *CDKN2A/B*, utilizing the Illumina EPIC Methylation Bead Chip, which incorporates integrated Copy Number Variation (CNV) profiling. A total of 45 glioblastomas were subjected to epigenome-wide DNA methylation profiling using the Illumina Methylation EPIC Bead Chip. These tumors were previously characterized both morphologically and molecularly. The resulting DNA methylation profiles were processed using the molecularneuropathology.org pipeline of the DKFZ. Glioblastomas were classified into three molecular subtypes: RTK I, RTK II, and mesenchymal (MES). Of the 45 patients, 14 (31%) were female and 31 (69%) were male. The mean patient age was 65 years. Of the thirteen patients with RTK I subtype, four (31%) were female and nine (69%) were male. The mean patient age was 62 years. Of the 15 patients with RTK II subtype, four (27%) were female and 11 (73%) were male. The mean patient age was 65 years. Of the 17 patients diagnosed with the MES subtype, six (35%) were female and 11 (65%) were male. The mean patient age was 66 years. Additional information regarding patient characteristics can be found in Table S1. DNA methylome analysis by EPIC arrays is a novel reliable approach in molecular glioma classification that is implemented in the current 2021 WHO classification of CNS tumors. 34 We found that EPIC analysis enabled to reveal *CDKN2A/B* loss (Figure 1A, Figure 1B) and retainment status (Figure 1C). Cut-off values were defined as suggested by Stichel *et al.* for analysis of Illumina Infinium Methylation bead chip data. 35

### Loss of* CDKN2A/B* predominantly occurs in RTK II glioblastoma

**Figure 2:**
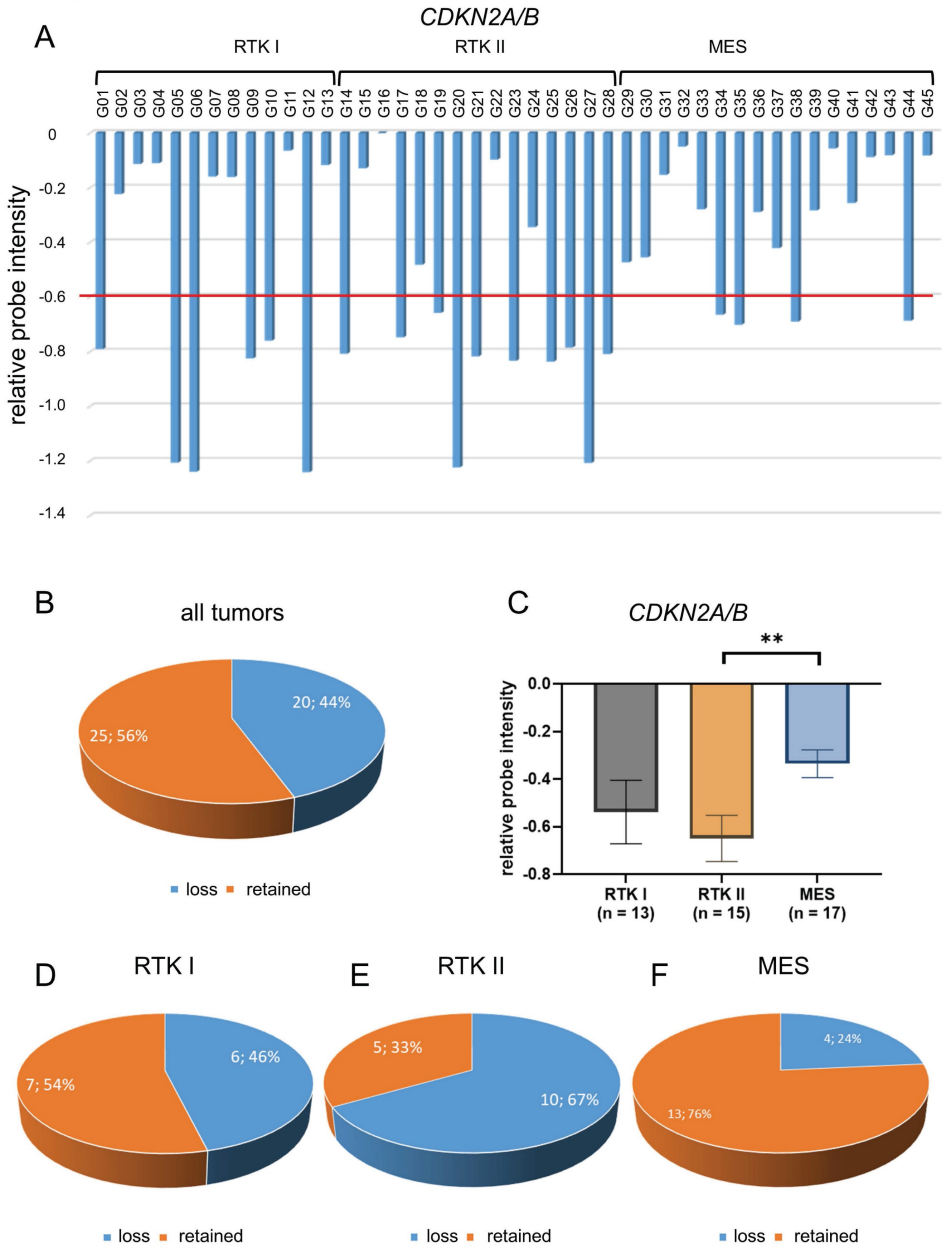
** Distribution of CDKN2A/B loss in glioblastomas.** CNV profiles of 45 glioblastomas CDKN2A/B were analyzed (A). Of all 45 glioblastomas CDKN2A/B loss was found in 20 samples (44%) (B). Statistical analysis CNV profiles shows significant loss of CDKN2A/B in glioblastoma of RTK II subtype compared with MES (C). Analysis of different subtypes showed that there is a loss of CDKN2A/B in six of thirteen glioblastomas of RTK I subtype (46%) (D), in ten of fifteen glioblastomas of RTK II subtype (67%) (E), and in four of seventeen glioblastomas of MES subtype (24%) (F). Indicated are mean ± SEM; ** p < 0.01.

The analysis of CDKN2A/B status was conducted on all 45 glioblastomas, employing the method of relative probe intensity (Figure 2A). Regardless of the molecular subtype, *CDKN2A/B* loss was found in 20 tumors (44%) (Figure 2B).

Detailed analysis showed that the mean *CDKN2A/B* chromosomal loss was -0.54 in RTK I subtype, -0.65 in RTK II subtype, and -0.34 in MES subtype scaled by relative probe intensities. Thereby, loss was significantly more frequent in RTK II subtype compared to the MES subtype (p < 0.01) (Figure 2C).

*CDKN2A/B* loss was found in six of thirteen glioblastomas of RTK I subtype (46%) (Figure 2D), in ten of 15 glioblastomas of RTK II subtype (67%) (Figure 2E), and in four of 17 glioblastomas of MES subtype (24%) (Figure 2F).

### Integrated analysis of *CDKN2A/B* loss and molecular glioblastoma hallmarks

Subsequently, an integrated analysis of CDKN2A/B loss and additional molecular glioblastoma hallmarks was conducted. The presence of TERT promoter mutations and MGMT promoter methylation has been observed.

**Figure 3:**
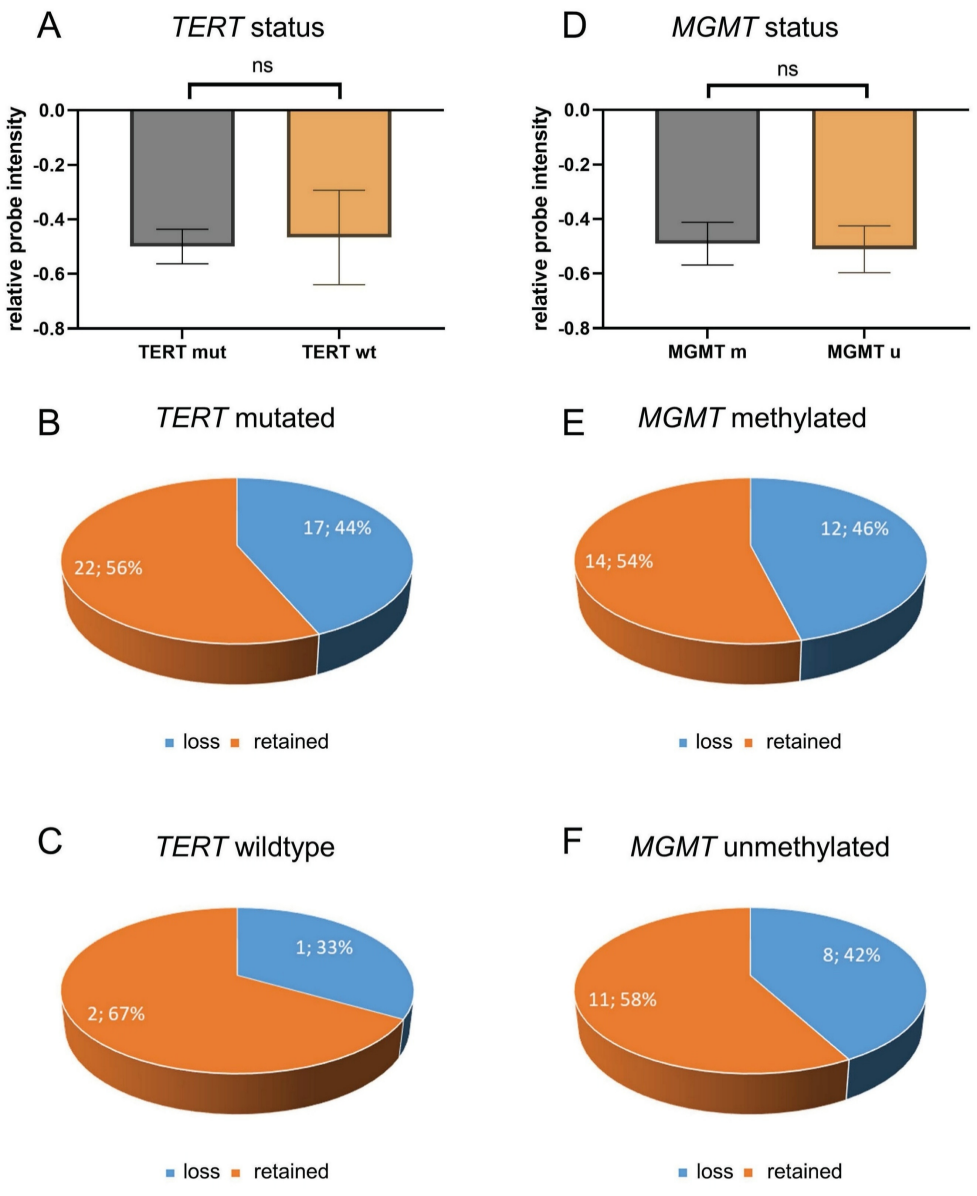
** Analysis of CDKN2A/B loss and TERT mutational and MGMT methylation status.** Analysis of CDKN2A/B loss and TERT promoter mutation status was performed in with known TERT mutation status (n=42), 39 cases showed TERT mutations while only three showed TERT wildtype status. Statistical analysis showed no association between CDKN2A/B loss and TERT promoter mutations (A) (p = 0.89). Of all 39 TERT mutated glioblastomas, 17 tumors showed CDKN2A/B loss and 22 tumors showed retained CDKN2A/B (B). Of all three analyzed TERT wildtype glioblastomas one case showed an CDKN2A/B loss while two cases showed retained CDKN2A/B (C). Analysis of MGMT promoter methylation was performed in all 45 samples. MGMT promoter was methylated in 26 cases and unmethylated in 19 cases. An analysis of CDKN2A/B loss and MGMT promoter methylation revealed that there is no association between CDKN2A/B loss and MGMT methylation status (D) (p = 0.86). Of all 26 MGMT methylated glioblastomas, twelve showed CDKN2A/B loss while 14 showed retained CDKN2A/B (E). Of all 19 MGMT unmethylated glioblastomas eight showed CDKN2A/B loss while 11 showed retained CDKN2A/B (F). Indicated are mean ± SEM. wt: wild type; m: methylated; u: unmethylated.

A comprehensive analysis was conducted on CDKN2A/B loss and TERT promoter mutation status in a total of 42 cases, with the caveat that the TERT mutation status was not available for three of these cases due to an insufficient amount of tissue (Table S1). Of all 42 glioblastomas with known *TERT* mutation status, *TERT* mutation were found in 39 cases (93%) while *TERT* wildtype status was found in only three cases (7%) (Table S1). Statistical analysis showed no association between *CDKN2A/B* loss and *TERT* promoter mutations (Figure 3A) (p = 0.89). Of all 39 *TERT* mutated glioblastomas, 17 tumors (44%) showed *CDKN2A/B* loss and 22 tumors (56%) showed retained *CDKN2A/B* (Figure 3B). Of the three *TERT* wildtype glioblastomas, one case (33%) showed *CDKN2A/B* loss while two cases (66%) showed retained *CDKN2A/B* (Figure 3C).

Further analysis was performed with regards to the *MGMT* promoter methylation status. *MGMT* promoter methylation status was available for all 45 cases (Table S1). *MGMT* promoter was methylated in 26 cases (58%) and unmethylated in 19 cases (42%) (Table S1). An analysis of *CDKN2A/B* loss and *MGMT* promoter methylation revealed that there is no association between *CDKN2A/B* loss and *MGMT* methylation status (Figure 3D) (p = 86). Of all 26 *MGMT* methylated glioblastomas, twelve showed *CDKN2A/B* loss while 14 showed retained *CDKN2A/B* (Figure 3E), and of all 19 *MGMT* unmethylated glioblastomas eight showed *CDKN2A/B* loss while 11 showed retained *CDKN2A/B* (Figure 3F).

## Discussion

Glioblastomas are the most aggressive primary brain tumors in adults. 36, 37 One crucial hallmark in glioblastoma therapy was the identification of *MGMT* promoter methylation as a predictive factor for improved response to treatment with the alkylating agent temozolomide and prolonged overall survival. 2-5

A novel promising biomarker in glioblastoma therapy may now be the detection of chromosomal loss of *CDKN2A/B*. The *CDKN2A* is a frequently inactivated tumor suppressor gene, e.g. it is the most commonly inactivated tumor suppressor gene in melanoma. 10 *CDKN2A* encodes the p16 and p14 proteins. 10 Inactivation of the gene group *CDKN2A/B* is a phenomenon that can be found in various malignancies, such as melanoma, 10 and non-small lung cancer. 38, 39 Common mechanisms of inactivation include somatic mutations, promoter methylation and chromosomal losses. 10, 22, 40

Downregulation of *CDKN2A* has been demonstrated to result in a number of significant biological effects, including decreased cellular viability, enhanced drug resistance, increased cellular self-renewal capacity, and altered expression of markers of pluripotency. 40 Changes in *CDKN2A* expression may thus be therapeutically relevant and a prognostic marker in patients with glioblastomas. 40 Here, we assessed *CDKN2A/B* chromosomal loss using the CNV profiles that are generated by applying the Infinium Methylation EPIC Bead Chip array. 34 We found that loss of *CDKN2A/B* is found in RTK II glioblastomas (Figure 2C). Despite the analysis of a sizable cohort, the validity of the results could be enhanced by verifying them in additional cohorts, such as the TCGA dataset in follow-up studies.

The importance of *CDKN2A/B* chromosomal loss has since already been established as precision medicine target in other malignancies, such as melanomas, 10 non-small lung cancer, 38, 39 and urothelial carcinoma 41 by reconstituting the tumor suppressing effect of *CDKN2A/B*. 10

Clinically, homozygous deletion of *CDKN2A/B* is highly relevant in terms of prognosis and as potential biomarker. 10, 23, 38-42

Loss of *CDKN2A/B* confers with aggressive biological behavior and poor prognosis in gliomas. This alteration disrupts key tumor suppressor pathways, notably the p16^INK4a^-CDK4/6-RB and the p14^ARF^-MDM2-p53 axes, leading to uncontrolled cell cycle progression and impaired apoptotic signaling. 10, 11 In IDH-mutant astrocytomas, *CDKN2A/B* deletion has been incorporated into the 2021 WHO CNS tumor classification as a criterion for grade 4 designation, even in the absence of necrosis or microvascular proliferation, underscoring its prognostic weight. 1 A recent study by Funakoshi *et al.* demonstrated that there is also a significant difference in overall survival of glioblastoma patients with unmethylated *MGMT* promoter: 23 patients with *CDKN2A* homozygous deletion showed median overall survival of 14.7 months while patients without *CDKN2A* homozygous deletion showed median overall survival of 16.9 months (p = 0.0129). 23 The discrepancy in outcomes between patients with and without *CDKN2A* homozygosis was particularly pronounced, with a median overall survival of 10.1 months in the former group and 15.6 months in the latter. This finding underscores the significance of *CDKN2A* homozygosis in predicting patient survival outcomes, particularly in the context of bevacizumab treatment (p = 0.0351). 23

Therapeutically, *CDKN2A/B* deletion presents actionable vulnerabilities. Recent studies have demonstrated the critical importance of identifying the *CDKN2A/B* chromosomal status in the provision of personalized patient care. 10, 38-42 Even though *CDKN2A/B* does not per se function as a potential molecular target, there is the potential for enhancing treatment outcomes through the restoration of the tumor-suppressing capabilities of *CDKN2A/B*. 10, 38-42 The findings indicate the possibility of innovative therapeutic methods for the management of brain tumors, thus facilitating the progress of precision medicine for glioblastoma patients by means of the therapeutic reconstitution of the tumor-suppressing effect of *CDKN2A/B* as it is already tried in other tumors, such as melanoma. 10 Minami *et al.* performed integrated analysis of the lipidome of glioblastoma and showed that *CDKN2A* deletions remodel the glioblastoma lipidome, i.e. leading to a redistribution of oxidizable polyunsaturated fatty acids into distinct lipid compartments. 42 Consequently, glioblastoma with *CDKN2A* deletion has been shown to exhibit elevated levels of lipid peroxidation, which results in the selective priming of tumors for ferroptosis. 42 These data emphasize a therapeutically exploitable link in glioblastoma between the phenomenon of recurring molecular lesions and an altered lipid metabolism. 42

CDK4/6 inhibitors, such as palbociclib, ribociclib, and abemaciclib, have shown promise in tumors and glioma models by restoring cell cycle control. 10, 17, 38-45 These agents may be particularly effective in tumors with intact *RB1* and concurrent *CDKN2A/B* loss, although clinical trials are needed to validate efficacy. 17, 40-42, 44, 45 Furthermore, *CDKN2A/B* deletions frequently co-occur with MTAP loss, which creates a metabolic vulnerability exploitable via synthetic lethality. 46-48 MTAP-deficient tumors accumulate methylthioadenosine (MTA), a PRMT5 inhibitor, sensitizing them to PRMT5 and MAT2A inhibitors. 46-48 Immunotherapeutic relevance is also emerging. *CDKN2A/B*-deleted gliomas exhibit altered immune microenvironments. 49-51 This may influence responsiveness to immune checkpoint inhibitors (ICIs). 49-51 While gliomas have historically shown limited response to ICIs, molecular stratification based on *CDKN2A/B* status may help identify subgroups with enhanced susceptibility, especially when combined with therapies that modulate the tumor microenvironment. 49-51

In summary, CDKN2A/B deletion not only serves as a prognostic marker but also represents a gateway to precision oncology, offering multiple therapeutic entry points through cell cycle inhibition, metabolic targeting, and immunomodulation. Our findings demonstrate that Infinium EPIC Bead Chip analysis, which is routinely applied in molecular brain tumor classification, 34 is a reliable technique for detection of *CDKN2A/B* chromosomal losses. In addition, our research has revealed that *CDKN2A/B* loss is a phenomenon that occurs predominantly in glioblastoma of the molecular subgroup RTK II.

## Supplementary Material

Supplementary Table S1. Details on glioblastoma samples and relative probe intensities.
