# Supplementary material for: Loss of CDKN2A/B is a Hallmark of RTK II Glioblastomas
Source: J Cancer. 2026 Jan 1;17(1):1–9. doi: 10.7150/jca.122609 (PMC12719383; doi:10.7150/jca.122609)
Supplement: Supplementary file 1 — Supplementary Table S1. Details on glioblastoma samples and relative probe intensities. [file jcav17p0001s1.pdf]

Table S1. Details on glioblastoma samples and relative probe intensities. Indicated are details on analyzed samples including age, sex, CDKN2A/B analysis and molecular genetic hallmarks. rpi: relative probe intensity, n.a.: not available, wt: wildtype, u unmethylated, m: methylated, d: deleted, n: not deleted.

ID	Age (y)	Sex	Diagnosis	Grade	Subtype	*IDH1*	*IDH2*	*TERT*	*MGMT*	CDKN2A/B	CDKN2A/B (rpi)
G01	71	m	Glioblastom IDH Wildtype	4	RTK I	wt	wt	C228T	m	loss	-0,79
G02	77	m	Glioblastom IDH Wildtype	4	RTK I	wt	wt	C228T	u	retained	-0,223
G03	45	f	Glioblastom IDH Wildtype	4	RTK I	wt	wt	C228T	m	retained	-0,113
G04	72	m	Glioblastom IDH Wildtype	4	RTK I	wt	wt	C228T	m	retained	-0,11
G05	63	f	Glioblastom IDH Wildtype	4	RTK I	wt	wt	C250T	m	loss	-1,206
G06	51	m	Glioblastom IDH Wildtype	4	RTK I	wt	wt	C250T	m	loss	-1,239
G07	76	f	Glioblastom IDH Wildtype	4	RTK I	wt	wt	wt	m	retained	-0,159
G08	66	m	Glioblastom IDH Wildtype	4	RTK I	wt	wt	C228T	u	retained	-0,161
G09	59	m	Glioblastom IDH Wildtype	4	RTK I	wt	wt	C228T	u	loss	-0,824
G10	61	m	Glioblastom IDH Wildtype	4	RTK I	wt	wt	wt	u	loss	-0,759
G11	53	f	Glioblastom IDH Wildtype	4	RTK I	wt	wt	C228T	m	retained	-0,064
G12	55	m	Glioblastom IDH Wildtype	4	RTK I	wt	wt	C228T	u	loss	-1,241
G13	62	m	Glioblastom IDH Wildtype	4	RTK I	wt	wt	C228T	u	retained	-0,117
G14	52	m	Glioblastom IDH Wildtype	4	RTK II	wt	wt	C228T	u	loss	-0,807
G15	62	m	Glioblastom IDH Wildtype	4	RTK II	wt	wt	C250T	m	retained	-0,129
G16	32	m	Glioblastom IDH Wildtype	4	RTK II	wt	wt	C228T	u	retained	0,021
G17	75	f	Glioblastom IDH Wildtype	4	RTK II	wt	wt	C250T	u	loss	-0,747
G18	77	f	Glioblastom IDH Wildtype	4	RTK II	wt	wt	wt	m	retained	-0,482
G19	77	f	Glioblastom IDH Wildtype	4	RTK II	wt	wt	C228T	u	loss	-0,658
G20	59	m	Glioblastom IDH Wildtype	4	RTK II	wt	wt	C250T	u	loss	-1,223
G21	64	m	Glioblastom IDH Wildtype	4	RTK II	wt	wt	C228T	m	loss	-0,817
G22	70	f	Glioblastom IDH Wildtype	4	RTK II	wt	wt	C250T	m	retained	-0,097
G23	42	m	Glioblastom IDH Wildtype	4	RTK II	wt	wt	C250T	m	loss	-0,833
G24	79	m	Glioblastom IDH Wildtype	4	RTK II	wt	wt	C228T	u	retained	-0,344
G25	79	m	Glioblastom IDH Wildtype	4	RTK II	wt	wt	n.a.	m	loss	-0,836
G26	58	m	Glioblastom IDH Wildtype	4	RTK II	wt	wt	C250T	m	loss	-0,784
G27	72	m	Glioblastom IDH Wildtype	4	RTK II	wt	wt	C228T	m	loss	-1,207
G28	83	m	Glioblastom IDH Wildtype	4	RTK II	wt	wt	C228T	u	loss	-0,808
G29	55	m	Glioblastom IDH Wildtype	4	MES	wt	wt	C228T	u	retained	-0,473
G30	66	m	Glioblastom IDH Wildtype	4	MES	wt	wt	C228T	m	retained	-0,455
G31	64	m	Glioblastom IDH Wildtype	4	MES	wt	wt	C250T	m	retained	-0,153
G32	78	m	Glioblastom IDH Wildtype	4	MES	wt	wt	C228T	m	retained	-0,049
G33	74	m	Glioblastom IDH Wildtype	4	MES	wt	wt	C250T	m	retained	-0,279
G34	65	f	Glioblastom IDH Wildtype	4	MES	wt	wt	C228T	m	loss	-0,665
G35	66	m	Glioblastom IDH Wildtype	4	MES	wt	wt	C228T	m	loss	-0,701
G36	63	f	Glioblastom IDH Wildtype	4	MES	wt	wt	C250T	m	retained	-0,289
G37	79	f	Glioblastom IDH Wildtype	4	MES	wt	wt	C228T	u	retained	-0,421
G38	67	f	Glioblastom IDH Wildtype	4	MES	wt	wt	C250T	m	loss	-0,69
G39	72	f	Glioblastom IDH Wildtype	4	MES	wt	wt	C250T	u	retained	-0,283
G40	38	m	Glioblastom IDH Wildtype	4	MES	wt	wt	n.a.	u	retained	-0,056
G41	85	m	Glioblastom IDH Wildtype	4	MES	wt	wt	C228T	m	retained	-0,256
G42	76	m	Glioblastom IDH Wildtype	4	MES	wt	wt	C228T	m	retained	-0,088
G43	55	m	Glioblastom IDH Wildtype	4	MES	wt	wt	C228T	u	retained	-0,081
G44	55	m	Glioblastom IDH Wildtype	4	MES	wt	wt	n.a.	m	loss	-0,687
G45	64	f	Glioblastom IDH Wildtype	4	MES	wt	wt	C228T	u	retained	-0,082
